# Mid-term outcome comparing temporary K-wire fixation versus PDS augmentation of Rockwood grade III acromioclavicular joint separations

**DOI:** 10.1186/1756-0500-2-84

**Published:** 2009-05-09

**Authors:** Bernd A Leidel, Volker Braunstein, Susann Pilotto, Wolf Mutschler, Chlodwig Kirchhoff

**Affiliations:** 1 Department of Traumatology and Orthopaedic Surgery, Ludwig-Maximilians-University Munich, Munich, Germany; 2 AO Foundation, AO Research Institute, Davos, Switzerland; 3 Department of Orthopaedics and Sports Medicine, Technical University Munich, Munich, Germany; 4 Klinikum der Universität München, Chirurgische Klinik und Poliklinik – Innenstadt, Nußbaumstraße 20, 80336 München, Germany

## Abstract

**Backround:**

The treatment of acute acromioclavicular (AC) joint injuries depends mainly on the type of the dislocation and patient demands. This study compares the mid term outcome of two frequently performed surgical concepts of Rockwood grade III AC joint separations: The temporary articular fixation with K-wires (TKW) and the refixation with an absorbable polydioxansulfate (PDS) sling.

**Findings:**

Retrospective observational study of 86 patients with a mean age of 37 years underwent either TKW (n = 70) or PDS treatment (n = 16) of Rockwood grade III AC joint injuries. Mid term outcome with a mean follow up of 3 years was measured using a standardized functional patient questionnaire including Constant score, ASES rating scale, SPADI, XSMFA-D and a pain score. K-wire therapy resulted in significantly better functional results expressed by Constant score (88 ± 10 vs. 73 ± 18), ASES rating scale (29 ± 3 vs. 25 ± 5), SPADI (3 ± 9 vs. 9 ± 13), XSMFA-D function (13 ± 2 vs. 14 ± 3), XSMFA-D impairment (4 ± 1 vs. 6 ± 2) and pain score (1 ± 1 vs. 2 ± 2).

**Conclusion:**

Either temporary K-wire fixation and PDS sling enable good or satisfying functional results in the treatment of Rockwood grade III AC separations. However functional outcome parameters indicate a significant advantage for the K-wire technique.

## Introduction

Approximately 9–12% of injuries to the shoulder girdle involve the acromioclavicular (AC) joint, mostly in young adults, especially in the young athletic patient population. Five times more men than women are concerned and incomplete AC joint dislocations are twice as frequent as complete ones [1]. While there is widespread agreement that non-operative treatment is recommended for grade I and II lesions, there is still an ongoing controversy about treatment of grade III AC joint separations according to Rockwood, especially for patients with high demands regarding the shoulder function [2-11]. Conservative therapy of grade III AC joint injury is widespread and many satisfying results are published [12,13]. However, some authors have reported residual symptoms of pain and weakness in up to 50% of non-operatively treated patients in cases of Rockwood grade III AC joint injuries [14,15].

Although various operative techniques have been proposed in recent literature, it remains unclear which procedure provides better functional outcome. Hence the aim of the present study was to compare the functional outcome over the years of two frequently performed surgical procedures following acute Rockwood grade III AC joint injuries in patients with high demands regarding shoulder function: the temporary fixation of the AC joint using rigid Kirschner wires (TKW) and the non-rigid fixation with an absorbable polydioxansulfate (PDS) sling.

## Patients and methods

In this retrospective study we compared the outcome of two different surgical repair techniques in patients consecutively treated at our orthopaedic department between 2002 and 2004 suffering from acute (<2 weeks old) injury. Inclusion criteria were lesion Rockwood grade III and high demands regarding shoulder function of the dominant arm in recreational sport activities [1,10]. Patients with signs of osteoarthritis, history of previous surgical procedure, injury to the AC joint and associated injuries to the acromion, coracoid or clavicle were excluded.

Following thorough standardized patient information, allocation to ether temporary fixation of the AC joint with K-wires (TKW) or refixation of the clavicle with an absorbable polydioxansulfate (PDS) sling was performed. The operative procedure was done in general anaesthesia and beach chair position with the injured limb freely mobile. Both applied surgical techniques were performed as previously published [6]. Using a vertical incision over the lateral clavicle toward the coracoid process, the AC joint including the articular surfaces, disk and ligaments were examined. After reducing the AC joint, 2 unthreaded 2 mm K-wires were inserted parallel consecutively from the lateral acromion into the clavicle in length each. To prevent proximal K-wire migration, the lateral pin ends were bent. The correct K-wire positions and AC joint position were verified with intra operative X-ray examination. Alternatively the subcoracoid passage was prepared by blunt dissection and the reduced AC joint stabilized with a 7.5 mm wide polydioxansulfate sling around the coracoid process and the clavicle. Additionally, in both groups the ruptured AC, coracoclavicular ligaments and deltotrapezoid fascia were reconstructed. A standardized rehabilitation program followed both procedures within 24 hours after surgery. The K-wires were removed in a standardized manner in local anaesthesia 6 weeks following their insertion.

For follow up, each patient was sent a standardized functional patient questionnaire. The questionnaire included the *Constant *score, *American Shoulder and Elbow Surgeons (ASES) *rating scale, *Shoulder Pain and Disability Index (SPADI), German Extra Short Musculoskeletal Function Assessment Questionnaire (XSMFA-D) *and an isolated pain score using the visual analogue scale (VAS). The applied ASES score was limited to the patient's self-assessment part with a maximum of 30 points achievable. The *German Extra Short Musculoskeletal Function Assessment Questionnaire (XSMFA-D) *is a score based on the *Short Musculoskeletal Function Assessment Questionnaire (SMFA) *established by American orthopaedic surgeons to evaluate musculoskeletal function from a patient's perspective in routine use [16]. The XSMFA-D is a 16-item version for routine assessment of functional capacity in patients with orthopaedic disorders. It takes into account the functional deficits and impairments due to inflammatory, degenerative and injury related causes. The XSMFA-D was validated as an appropriate short questionnaire for the evaluation of therapy results from the patient's perspective [17].

For statistical testing SPSS version 13.0 software (SPSS, Chigaco IL, USA) was employed. According to the distribution and sample size, for statistic evaluation the Mann-Whitney Rank Sum test was applied to analyze the differences in outcome between the two treatment groups at a significance level of p = 0.05.

## Results

The study collective included altogether 86 patients with a high physical activity level including regular recreational sport activities, like playing golf, tennis and swimming. They emphasized high demands concerning their shoulder function and had active employment relationships. Gender distribution was 93% male in the TKW group and 88% male in the PDS group. The average age at time of injury was 37.3 (SD ± 11.5) years. 70 patients underwent temporary articular fixation of the AC joint with K-wires (TKW), 16 patients the refixation of the clavicle with an absorbable polydioxansulfate (PDS) sling. The following up period was in average 4.2 (SD ± 2.5) years. There were no statistically significant differences between both groups regarding the patients' age and the following up period (Table 1).

**Table 1:** Patient characteristics

	**Age **(SD) [years]	**Follow Up **(SD) [years]
**TKW **(n = 70)	37.4 (± 11.1)	4.4 (± 2.6)

**PDS **(n = 16)	36.7 (± 13.5)	3.2 (± 2.2)

Mean age and follow up period; SD = standard deviation, TKW = temporary fixation Kirschner wires, PDS = absorbable polydiaxanone sling

In general, according to our standardized questionnaire including the Constant score, ASES rating scale, SPADI, XSMFA-D and the isolated pain score, patients in the TKW group achieved significantly better results compared to the PDS group (Table 2).

**Table 2:** Mean functional questionnaire results in points (Pts)

	**Constant **(SD) [Pts]	**ASES **(SD) [Pts]	**SPADI **(SD) [Pts]	**XSMFA-D **Function (SD) [Pts]	**XSMFA-D **Impairment (SD) [Pts]	**Pain **(SD) (0 = no pain, 10 = max. pain) [Pts]
**TKW **(n = 70)	87.8 (± 10.3)	28.5 (± 3.4)	2.5 (± 8.7)	12.5 (± 1.5)	4.4 (± 1.1)	0.5 (± 0.9)

**PDS **(n = 16)	73.0 (± 17.7)	25.4 (± 5.0)	9.4 (± 13.3)	14.0 (± 3.1)	6.0 (± 2.2)	1.9 (± 1.9)

**P**	< 0.001	< 0.001	< 0.001	0.028	0.004	0.004

Standard deviation (SD) and significance (p) between treatment groups; TKW = fixation Kirschner wires, PDS = absorbable polydiaxanone sling

**Figure 1:**
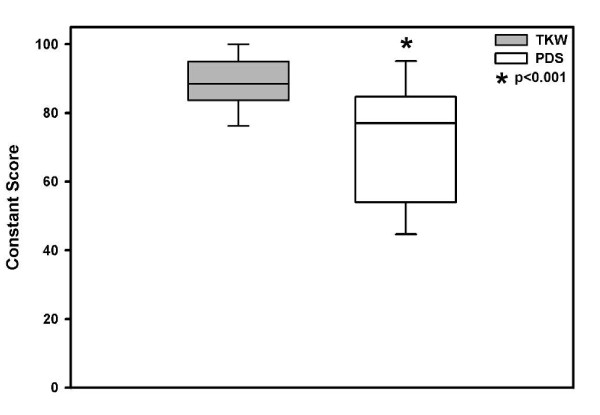
**Mean ± SD functional outcome Constant score significant better in the TKW group, but in both treatment groups "good"**.

In the TKW group the mean Constant score was 87.8 versus 73.0 points in PDS treated patients, with a significant difference between both treatment groups (p < 0.001). The results in both groups rated as good according to the score (Figure 1).

**Figure 2:**
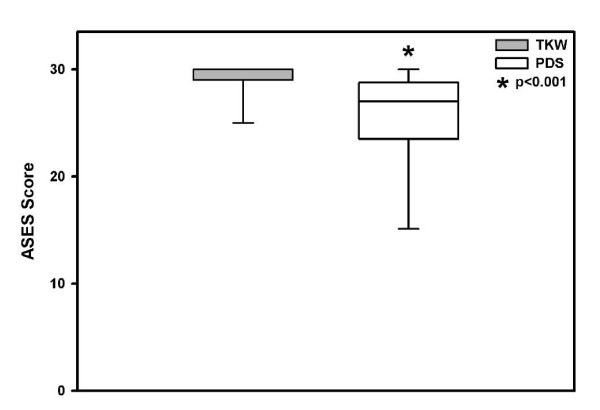
**Mean functional outcome ASES score significant better in the TKW group, but in both treatment groups "good"**.

The ASES rating scale showed out on mean in the TKW group 28.5 versus 25.4 points in the PDS group of maximally 30 points. The difference between both groups was significant (p < 0.001) (Figure 2).

**Figure 3:**
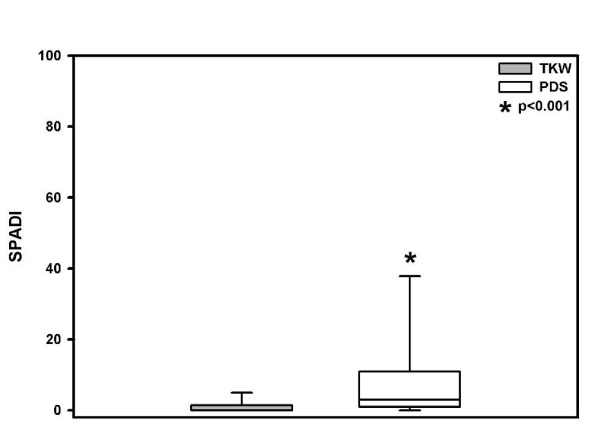
**Mean ± SD functional outcome SPADI score significant better in the TKW group, but in both treatment groups "good"**.

Applying the SPADI in the TKW group patients achieved significant different on mean 2.5 points versus 9.4 points in the PDS group (p < 0.001) (Figure 3).

**Figure 4:**
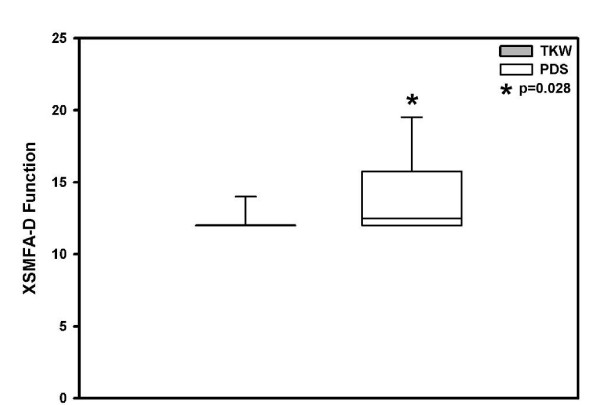
**Mean ± SD functional outcome XSMFA-D score significant better in the TKW group, but in both treatment groups "good"**.

**Figure 5:**
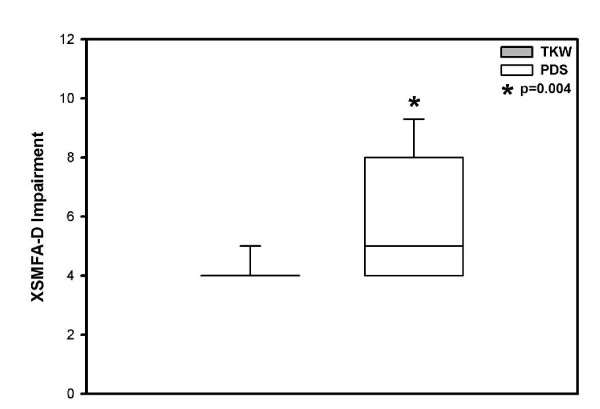
**Mean ± SD impairment XSMFA-D score significant better in the TKW group, but in both treatment groups "good"**.

The XSMFA-D function score on average the TKW treated patient group reached 12.5 versus 14.0 points (mean) in the PDS treated group with a significant difference (p = 0.028) (Figure 4). With regard to the XSMFA-D impairment score on average the TKW group reached 4.4 versus 6.0 points in the PDS group (p = 0.004), with a significant difference in both treatment groups (Figure 5).

The TKW group had also significantly less pain, with low pain complaints in both groups, ranging on average between 0.5 and 1.9, where 10 would be the subjective most severe pain (p = 0.004).

As for complications, 4% (n = 3) suffered pin migration in the TKW group and there was 44% (n = 7) complaining clinical loss of AC joint reduction by patients' perception in the PDS group. Infection was not observed in anyone of the overall 86 patients.

## Discussion

The treatment strategy for Rockwood grade III AC separations shows geographically fundamental differences. In a recent survey of members of the *American Orthopaedic Society for Sports Medicine (AOSSM) *and the *Accreditation Council for Graduate Medical Education (ACGME) *orthopaedic program residency directors, *Nissen et al. *published the results of the 664 respondents. More than 80% of them continue to treat uncomplicated type III AC joint separations non-operatively with just an arm sling for comfort. For surgical management, respondents recommended rigid stabilization of the AC joint during early postoperative rehabilitation in 80–82% of cases [18]. In contrast published *Bäthis et al. *a recent survey of German trauma units, reporting 84% of the 104 respondents preferred the surgical approach in grade III AC joint injuries. The most frequent operative techniques of choice were temporary K-wire fixation followed by PDS sling [19].

*Bäthis et al. *analyzed the published non-operative and surgical therapy results of grade III AC joint separations. A systematic literature search according to *Cochrane Collaboration *including the databases of *Medline *and the *National Library of Medicine *for the period of 1980 to 1999 revealed 370 relevant papers classified in randomized controlled trials, comparative retrospective studies and retrospective studies. Overall the major outcome for both operative and non-operative treatment was similar. The author concluded that non-operative treatment appears to be the method of choice unless the patient's preference is operative therapy [20].

Other authors recommend a treatment decision in a more nuanced light concerning anatomical fixation, taking into account the age, sport activity level, heavy manual labour and overhead working habits of the patient [2-11].

In central Europe recommended treatment for grade III AC joint injury is very often surgical for patients with a high demand regarding the shoulder function. Therefore we compared the two most frequently performed surgical methods. Up to now no clear benefits of one surgical technique over the other in grade III AC joint dislocations have been described [3,6,8,11,21,22].

Our results after reconstructing the AC joint with an absorbable polydioxansulfate (PDS) sling seems not to be favourable compared to the technique using K-wires for temporary fixation. This may be caused by loss of reduction in the PDS group with an incidence of 44%. Perhaps this is attributed to the observed up to 60% elongation of the PDS material [23-25]. Furthermore the K-wire is placed with minimal soft tissue damage, the PDS is typically placed in sling fashion around the coracoid and clavicle, therefore requiring large exposure, resulting in soft tissue damage.

However, the K-wire technique implies the risk of pin migration, observed in three patients. The migrated pins impended to perforate the skin lateral of the acromion and had to be removed one respectively two weeks earlier than planned. Nevertheless, there were no adverse effects regarding shoulder function. Furthermore the temporary K-wire technique always necessitates a second surgical procedure to remove the wires [12].

The limitations of this study include the different treatment group size, 70 versus 16. Nevertheless a statistical comparison of both groups may be permissible due to the normality distributed patient characteristics in each group (age, injury pattern and following up period). Furthermore the allocation between the two groups reflects the typical distribution in these mostly accomplished surgical procedures for grade III AC separations in central Europe [12,21].

Our study revealed that patient self-assessment questionnaires seem to correlate treatment results reliably, with better outcome scores in each test for the TKW treated group. In a recent study *Boehm et al. *proved the reliable and valid correlation between shoulder function and a Constant score based on patient self-assessment only, without clinical examination and radiographic assessment. The self-assessing patient may reflect the functional outcome best by critical self-evaluating, due to the very often very individual expected treatment results. Furthermore patients tend to downgrade their shoulder function when compared to physical examination results [26].

Radiographic evaluation was abandoned due to the frequent reported lack of correlation between abnormal findings and clinical outcome. Detectable persistent AC joint incongruence or posttraumatic arthritis seems not to correlate with functional treatment results. Residual AC dislocation does not imperatively lead to complaints and loss of strength [3,8,15,27,28]. *Greiner et al. *found radiographic signs of degenerative joint disease in 36% of cases, however those changes were not correlated with lower functional outcome score [3]. *Larsen et al. *gave an account of 43 conservatively treated patients with very good or good treatment results in 97% of patients despite of residual AC dislocation [8]. *Rawes et al. *found in all of the evaluated 30 patients residual AC joint dislocations or subluxations following 12.5 years after AC grade III injury, nevertheless 29 had a good functional outcome [15]. *Taft et al. *reported about a 35% rate of radiographic detectable posttraumatic AC joint arthritis in 127 patients. Though, this had no impact on the clinical outcome [27]. *Prokop et al. *reported a 24% incidence of residual AC joint subluxation and a 17% incidence of posttraumatic arthritis in 66 patients. Yet, these radiographic findings did not correlate with patient complaints [28].

## Conclusion

This study shows that surgical treatment with K-wires or PDS sling in acute Rockwood grade III AC joint injury enables promising functional outcome and low pain experience in the mid term. Furthermore the K-wire technique proves better functional results, concerning range of motion, strength and pain discomfort.

## Competing interests

The authors declare that they have no competing interests.

## Authors' contributions

BAL conceived the study, acted as primary physician conducting data acquisition, recruiting subjects, analyzed results and drafted the manuscript. VB assisted with initial study design, analysed results, helped with the statistic workup and helped draft the manuscript. SP assisted in testing the subjects, data acquisition, analysis and drafting the manuscript. WM assisted with initial study design, result analyzing and helped draft the manuscript. CK assisted with study design, second observer for data acquisition, result analyzing, statistical analysis and drafting of the manuscript. All authors read and approved the final manuscript.
